# Multi-level Quantum Mechanics and Molecular Mechanics Study of Ring Opening Process of Guanine Damage by Hydroxyl Radical in Aqueous Solution

**DOI:** 10.1038/s41598-017-08219-z

**Published:** 2017-08-10

**Authors:** Peng Liu, Qiong Wang, Meixing Niu, Dunyou Wang

**Affiliations:** 1 College of Physics and Electronics, Shandong Normal University, Jinan, 250014 China; 2 College of Chemistry, Shandong Normal University, Jinan, 250014 China

## Abstract

Combining multi-level quantum mechanics theories and molecular mechanics with an explicit water model, we investigated the ring opening process of guanine damage by hydroxyl radical in aqueous solution. The detailed, atomic-level ring-opening mechanism along the reaction pathway was revealed in aqueous solution at the CCSD(T)/MM levels of theory. The potentials of mean force in aqueous solution were calculated at both the DFT/MM and CCSD(T)/MM levels of the theory. Our study found that the aqueous solution has a significant effect on this reaction in solution. In particular, by comparing the geometries of the stationary points between in gas phase and in aqueous solution, we found that the aqueous solution has a tremendous impact on the torsion angles much more than on the bond lengths and bending angles. Our calculated free-energy barrier height 31.6 kcal/mol at the CCSD(T)/MM level of theory agrees well with the one obtained based on gas-phase reaction profile and free energies of solvation. In addition, the reaction path in gas phase was also mapped using multi-level quantum mechanics theories, which shows a reaction barrier at 19.2 kcal/mol at the CCSD(T) level of theory, agreeing very well with a recent *ab initio* calculation result at 20.8 kcal/mol.

## Introduction

The damage of DNA caused by reactive radicals may result in mutagenesis, cell death, and carcinogenesis. These reactive radicals can arise from ionizing radiation^1–7^, chemical compounds^6, 8^, or reactive oxygen species created by cellular metabolism^2, 5, 6^. The hydroxyl radical (•OH), one of the most reactive oxygen species, is a primary radical formed from the radiolysis of water or from a chemical source, and is the mediator of much of the DNA damage caused by ionizing radiation^9^. The main classes of •OH radical mediated DNA lesions include strand-breaks, altered bases, abasic sites, and DNA-protein cross-links. Among the DNA bases, guanine has the lowest oxidation potential and is the most easily oxidized of the nucleic acid bases; hence, guanine is the most susceptible to be attacked by oxidizing agents including hydroxyl radical^1, 6, 10, 11^.

One of the major DNA lesions under the •OH attack is 2,6-diamino-4-hydroxy-5-formamidopyrimidine (FAPyG)^1, 12–15^. Pulse radiolysis studies^1, 12–18^ found that •OH attacks C_8_ site of guanine and leads to 8-hydroxyguanine radical (8-OHGrad), where further oxidation leads to 8-oxo-7,8-dihydroguanine (8-oxoG) and FAPyG^1, 12–18^. There have been active studies on the formation of FAPyG both experimentally^4, 19–22^ and theoretically^23–41^.

Among the experimental studies^4, 19–22^, Steenken’s group^4^ found the rapid ring-opening process of 8-OHGrad to FAPyG in oxygen-free aqueous solution has a rate constant of 2 × 10^5^ s^−1^ at PH 4–9; Burgdorf and Carell^19^ reported that the FAPyG lesion is significantly more stable than previously expected; Arce’s group^20^ found that, in the low- and high-intensity radiation of guanine derivatives, the opening of the purine ring to form FAPyG products serves as the major products.

Using density function theory (DFT), the H_2_O (modeled by OH^−^) addition on the C8-site of the guanine is exothermic by −75.3 kcal mol^−1^ ^24^. Another DFT calculation shows that the thermodynamic energy of initial binding of •OH with guanine in a hydrated environment (or in the gas phase) is in the order of C8HO• > C4HO• > C5HO•^25^. Various DFT calculations were also performed to study the reaction pathways leading to 8-oxoG^26–28^. Molecular dynamics simulations based on ReaxFF force field on a large fragment of DNA were carried out to simulate DNA damage^29–31^. A hybrid QM/MM approach was carried out to study the hydrogen abstraction by OH radicals to form a guanine-based DNA residue in aqueous solution, where the OH radicals and the guanine base were treated with a plane-wave-based DFT^32^. In addition, different pathways for oxidation of guanine, and reduction potentials of intermediates in the oxidation of guanine were also explored using DFT/B3LYP level of theory with IEF-polarizable continuum solvation model (PCM)^33–36^ and SMD implicit solvation model^37–40^, respectively.

Regarding the ring-opening of 8-OHGrad to FAPyG reaction mechanism, Munk *et al*. mapped four reaction pathways for the ring opening reaction of 8-OHGrad to FAPyG using the DFT/B3LYP theory in gas phase and with IEF-PCM model in aqueous solution^33^. The direct ring opening of the 8-OHGrad to intermediate species formimidic acid radical appears in two pathways, which can either be reduced to formimidic acid and then undergo tautomerization to FAPyG, or be tautomerized to FAPyG radicals before being reduced to FAPyG. The barrier height of direct ring opening process as discussed above was calculated to be 19.5 kcal mol^−1^ in aqueous solution, 15.1 kcal mol^−1^ in the gas phase. However, a recent *ab initio* study with multiconfigurational MCSCF/DZP and MRPT2 levels of theory by Chaban *et al*.^41^ shows that barrier height for ring opening of the 8-OHGrad to formimidic acid radical is 21.4 and 20.8 kcal mol^−1^ respectively in the gas phase, which is much higher than the 15.1 kcal mol^−1^ calculated by Munk *et al*. in the gas phase. Thus, there is a big uncertainty in the activation barrier height calculated using different levels of theory in gas phase.

So, in this study, we want not only to investigate the ring opening process of hydroxyl radical attacking guanine in gas phase, but also to investigate its reaction pathway in its real environment: aqueous solution. Because earlier studies of guanine reactions mainly are based on the DFT level of theory, we want to not only calculate the reaction pathway at the DFT level of theory, but also calculate its accurate reaction pathway based on the more accurate CCSD(T) level of theory in both the gas phase and solution phase. In addition, most of studies of the guanine reactions in solution used an implicit or continuum solvation model, here we used an explicit SPC/E water model^42^ to treat the aqueous solution to reveal molecular level, ring-opening mechanism of •OH attacking guanine in aqueous solution. Therefore, in this work, we combined multi-level quantum mechanics theories and molecular mechanics (ML-QM/MM)^43–45^, to investigate the solvent effects on the guanine ring-opening mechanism under •OH radical attack, to map an accurate potential of mean force (PMF) at the more accurate CCSD(T)/MM level of theory. Furthermore, the direct calculated PMF at the CCSD(T)/MM level of theory was verified by comparing with the PMF derived using the gas-phase potential minimum energy path and free energies of solvation.

## Results and Discussion

**Figure 1:**
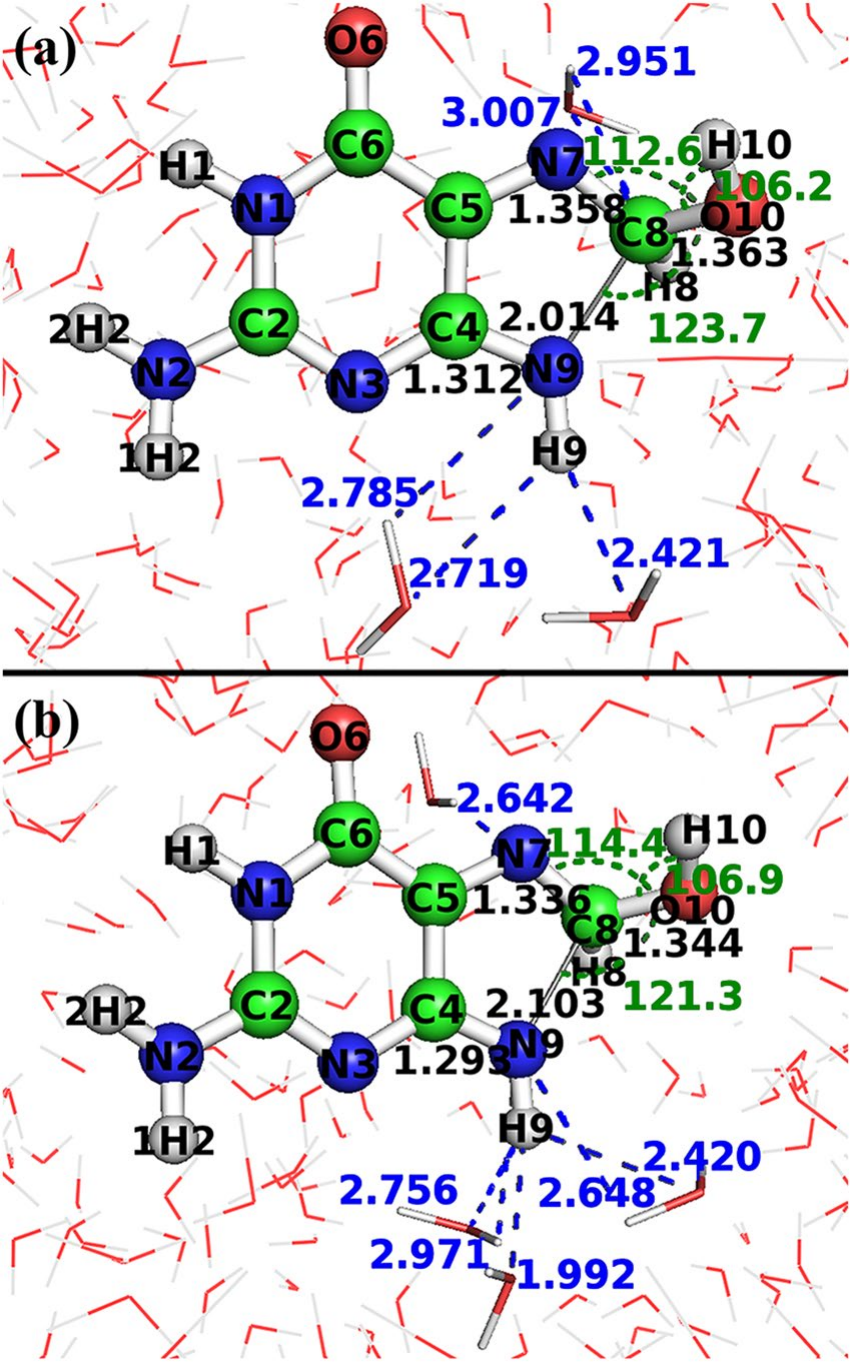
Comparison of transition states calculated with different basis sets in the solution phase. (**a**) Transition state obtained with aug-cc-pVDZ basis set, (**b**) transition state obtained with 6-311++G** basis set. Both structures are obtained at DFT/B3LYP level of theory; the indicated distances are in angstroms (bond lengths labeled in black and hydrogen bonds in blue) and angles in degrees (labeled in green).

Figure 1 shows the geometries of the transition states computed using the DFT/B3LYP method with the aug-cc-pVDZ and 6–311++G** basis sets respectively. This comparison shows that the two basis sets produce very similar results: the bond lengths of N7-C8, C8-O10, C8-N9 and N9-C4 are 1.358 Å, 1.363 Å, 2.014 Å and 1.312 Å with the aug-cc-pVDZ basis, while they are 1.336 Å, 1.344 Å, 2.103 Å and 1.293 Å with the 6–311++G** basis, with an unsigned mean difference less than 0.04 Å; the angles of ∠N7C8O10, ∠N9C8O10 and ∠C8O10H10 are 112.6°, 123.7° and 106.2° with the aug-cc-pVDZ basis, while 114.4°, 121.3° and 106.9° with the 6–311++G** basis, with an unsigned mean difference of 1.6°; the dihedral angles of ∠C5N7C8O10, ∠C5N7C8H8, ∠N7C8O10H10, ∠C8H8N9H9 and ∠C8H8O10H10 are −134.1°, 86.1°, 8.5°, −145.5° and −34.1° with the aug-cc-pVDZ basis, while −132.3°, 78.6°, 7.8°, −147.1° and −25.1° with the 6–311++G** basis, with an unsigned mean difference of 4.1°. There are five weak hydrogen bonds in Fig. 1a, with an average distance of 2.777 Å, six weak hydrogen bonds in Fig. 1b, with an average distance of 2.572 Å.

### Stationary points along the reaction path

**Figure 2:**
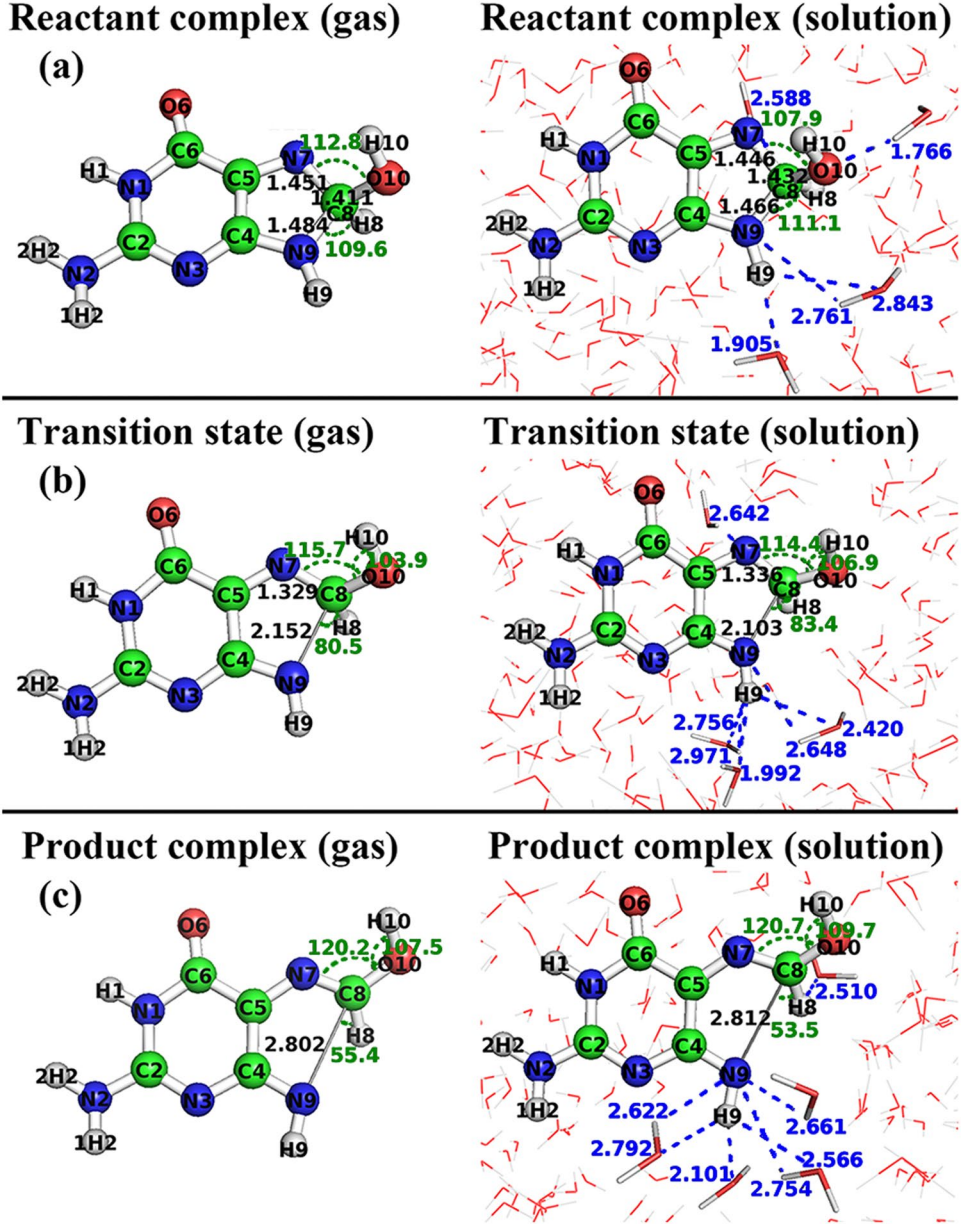
Comparisons of the stationary points for the ring opening reaction of 8-OHGrad to formimidic acid radical in gas phase and in aqueous solution. The indicated distances are in angstroms (bond lengths labeled in black and hydrogen bonds in blue) and angles in degrees (labeled in green).

The stationary points along the nudged elastic band (NEB) reaction pathway^46^ both in gas phase and in solution phase presented here were calculated with DFT/B3LYP/6–311++G** level of theory. The structures of reactant complex in gas phase and in solution phase are compared in Fig. 2a. The biggest differences of these two structures are not in their bond distances and bending angles, but in their dihedral angles. For example, there is only 0.021 Å difference of the distances between the •OH radical and the C8 atom: it is 1.411 Å in the gas phase; it is only increased to 1.432 Å due to the water interference. And the bending angles of ∠N7C8O10, ∠N7C8H8, and ∠H8C8N9 are 112.8°, 111.5°, and 109.6° in gas phase, while 107.9°, 113.6°, and 111.1° in solution phase. However, the dihedral angles of ∠C5N7C8O10, ∠C5N7C8H8, and ∠C8H8N9H9 are −123.4°, 118.1°, and −130.1° in the gas phase, while they are −115.8°, 127.7°, and −138.9° respectively in the solution phase. These differences are mainly caused by the presence of hydrogen bonds, as can be seen that there is a strong hydrogen bond at 1.766 Å in the solution phase between the •OH and its adjacent water molecule; there are also four weak hydrogen bonds, one from C8H8···H_2_O and three from N9H9···H_2_O with an average distance, 2.524 Å. As a result, the interactions between the 8-OHGrad and its surrounding water molecules, especially the hydrogen bonds formed between them, cause the geometry difference from the gas phase.

The structures of transition state from both gas phase and solution phase, are shown in Fig. 2b, which were confirmed by numerical frequency calculations with single imaginary frequencies of 236.2*i* cm^−1^ and 280.9*i* cm^−1^ respectively. Compared to the reactant complexes, the distance between C8 and N9 increases while the N7 and C8 bond length decreases. For example, in the solution phase, the C8 and N9 distance increases from 1.466 Å in reactant complex to 2.103 Å in transition state while the N7 and C8 distance decreases from 1.446 Å in reactant complex to 1.336 Å in transition state. Again, the biggest differences of these two transition states still remain in their dihedral angles. In solution, the bending angles of ∠N7C8O10, ∠N9C8H8 and ∠C8O10H10 are 114.4°, 83.4° and 106.9°, while 115.7°, 80.5° and 103.9° in the gas phase. In solution, the dihedral angles of ∠C5N7C8O10, ∠C5N7C8H8, ∠N7C8O10H10, and ∠C8H8O10H10 are −132.3°, 78.6°, 7.8°, and −25.1°, while they are −136.5°, 67.6°, 5.2°, and −20.2° in gas phase. In addition, six weak hydrogen bonds were formed with an average distance of 2.572 Å, where one interacts with C8H8 and five interact with N9H9 in the solution phase.

The product complexes, formimidic acid radical, for both in gas phase and solution phase, are shown in Fig. 2c. At the product state, the right ring of 8-OHGrad has been damaged and broken at C8N9 site by the •OH radical. The atom O10 keeps on moving to the upper right with an increasing angle ∠N7C8O10, from 107.9° in the reactant complex, to 120.7° in the product complex, and the atoms C8, H8, O10 and H10 are almost coplanar with a dihedral angle 0.1°. The broken bond of C8-N9 almost have the same distance, 2.802 Å in gas phase and 2.812 Å in aqueous solution. The angles of ∠N9C8H8 and ∠C8O10H10 are 53.5° and 109.7° in aqueous solution, while 55.4° and 107.5° in the gas phase. Again, the biggest differences between the two structures still exist in their torsion angles: the dihedral angles of ∠C5N7C8O10, ∠C5N7C8H8, and ∠C8H8N9H9 are −163.2°, 19.0°, and −146.9° in solution phase, while they are −170.4°, 11.6°, and −170.1° in gas phase. Furthermore, there are seven weak hydrogen bonds with C8H8···H_2_O and N9H9···H_2_O in solution phase, with an average distance of 2.572 Å.

In short, compared to the structures of stationary points in gas phase, the aqueous solution affects the geometry mainly through the torsion angles, different from our previous investigation of S_N_2 reaction^47, 48^ that interactions between the solvent and solute have big impact on the bond lengths and bending angles of the corresponding geometries too.

### Evolutions of geometry along the NEB reaction pathway

**Figure 3:**
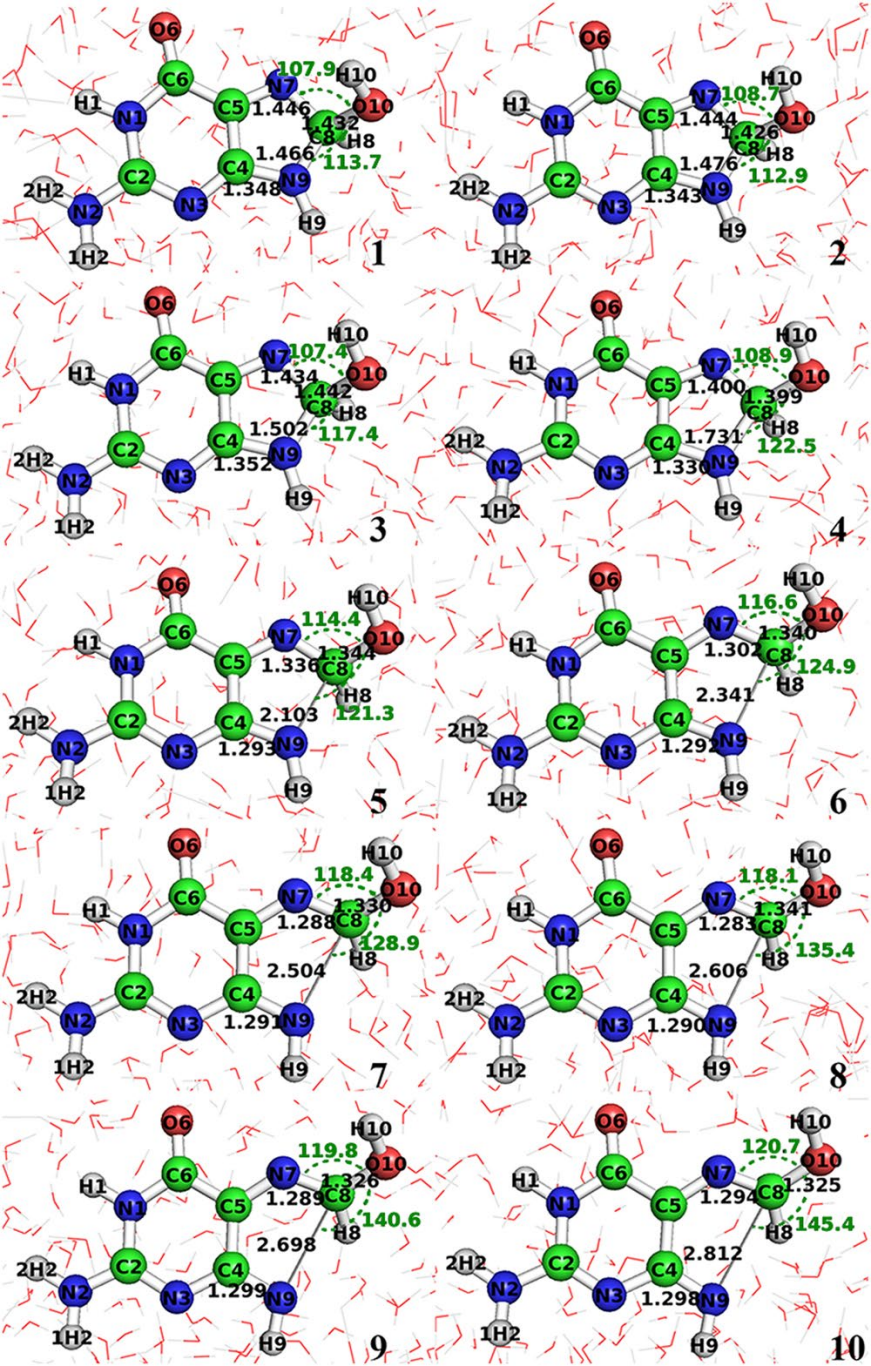
The structures of 10 snapshots along the NEB reaction pathway for the ring opening reaction of 8-OHGrad to formimidic acid radical in aqueous solution. No. 1 is the structure of reactant complex, No. 5 transition state, and No. 10 product complex. The indicated distances are in angstroms (bond lengths labeled in black and hydrogen bonds in blue) and angles in degrees (labeled in green).

In order to show the detailed, atomic-level ring-opening reaction mechanism, ten snapshots along the NEB reaction pathway^46^ are plotted in Fig. 3. Figure 3.1 is the reactant structure, Fig. 3.5 is the transition state structure and Fig. 3.10 the product structure. The main feature of ring-opening reaction mechanism is the breaking of C8-N9 bond, which is 1.466 Å at reactant complex, increased to 2.103 Å at transition state, and finally to 2.812 Å at product complex. Consequently, the atoms N7, C4 and O10 which are connected to C8 and N9 change their corresponding bond lengths too. For example, the N7-C8 bond decreases from 1.446 Å to 1.294 Å in the evolution process; the C4-N9 bond decreases from 1.348 Å to 1.298 Å; and the C8-O10 bond decreases from 1.432 Å to 1.325 Å. Furthermore, there are big changes in their bending and dihedral angles. For instance, the dihedral angle of ∠N7C8O10H10 decreases from 25.7° in reactant complex to 7.8° in transition state, then to 2.1° in product complex; the dihedral angle of ∠C8H8N9H9 increases from −138.9° in reactant complex to −147.1° in transition state, then to −146.9° in product complex; and the dihedral angle of ∠C8H8O10H10 decreases from −43.5° in reactant complex to −25.1° in transition state, then to 0.1° in product complex. In addition, the angle of ∠N7C8O10 increases from 107.9° in reactant complex to 114.4° in transition state, then to 120.7° in product complex; and the angle of ∠N9C8O10 increases from 113.7° in reactant complex to 121.3° in transition state, then to 145.4° in product complex.

In short, the 8-OHGrad ring-open process is not only the breaking of C8-N9 bond appearing in the evolution process, but also the big changes of the bending and torsion angles related to the C8 and N9 atoms. These angles are changed significantly along the reaction process.

### Potentials of mean force and solvent contributions

**Figure 4:**
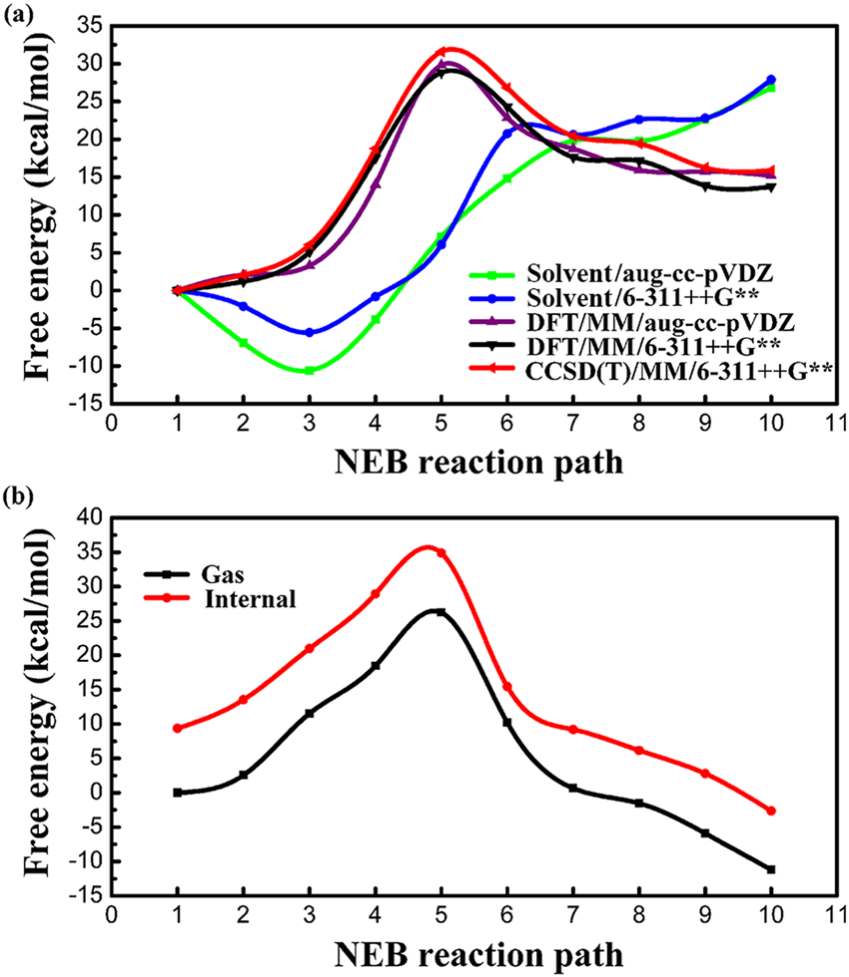
Potentials of mean force and solvent energy contributions along the NEB reaction path. (**a**) Potentials of mean force calculated at DFT/MM and CCSD(T)/MM levels of theory and solvent energy contribution with the reactant state as a reference point, (**b**) comparison between “gas phase” and solute internal energies along the NEB reaction pathway under the CCSD(T)/MM/6-311++G** level of theory using the gas-phase energy of the reactant complex as a reference point.

The PMFs of ring-opening reaction of 8-OHGrad to formimidic acid radical along the NEB reaction pathway in aqueous solution are plotted in Fig. 4a at both the DFT/MM and CCSD(T)/MM levels of theory, respectively. The results show that the free-energy barrier height is 28.8 kcal mol^−1^ at DFT/MM level of theory, while 31.6 kcal mol^−1^ with the CCSD(T)/MM level of theory, both with the 6–311++G** basis set. The free reaction energy is 15.9 kcal mol^−1^ at CCSD(T)/MM level of theory. The solvent energy contributes 6.0 kcal mol^−1^ to free energy barrier height and 27.9 kcal mol^−1^ to free reaction energy. Though the aug-cc-pVDZ basis set was unable to be utilized to shift to CCSD(T) level calculation, we also show the PMF with aug-cc-pVDZ basis set for DFT/MM level of theory. Figure 4a shows that the PMF of the DFT/MM calculations with 6–311++G** basis agrees very well with the one with aug-cc-pVDZ basis set, which again proves that the DFT/MM with 6–311++G** calculations gives reliable results to be shifted to the CCSD(T)/MM level of calculations.

In the study of assessing electronic structure model chemistries for chemical reaction barrier heights by Truhlar and coworkers^49^, the aug-cc-pVDZ basis set is recommended for DFT calculations when larger basis sets are affordable. Moreover, the number of basis function of the aug-cc-pVDZ basis set is 354 for the QM subsystem, which is bigger than one of the 6–311++G** basis set, 318; therefore, the former basis set should give more accurate results than the latter for the CCSD(T)/MM calculation. However, the huge CPU memory requirement with the aug-cc-pVDZ basis calculation at the CCSD(T)/MM level cannot be fullfilled by our computing resources, so we have to settle the calculation with the smaller 6–311++G** basis set.

To show the solvent polarization effect, we compare the ‘gas-phase’ and internal QM/MM energy in Fig. 4b (both were calculated with the CCSD(T)/MM/6–311++G** level), with ‘gas-phase’ reactant as the reference point. Here the ‘gas-phase’ energy is obtained with the same 10 beads on the PMF reaction path without solvent-solute interaction and MM contribution; the internal QM/MM energy is obtained excluding the MM energy contribution. The results show that the polarization effect contributes 9.4 kcal mol^−1^ to reactant state, 8.7 kcal mol^−1^ to transition state, and 8.6 kcal mol^−1^ to product state. As a result, the net polarization effect contributes −0.7 kcal mol^−1^ to transition state, and −0.8 kcal mol^−1^ to product state. Therefore, the aqueous solution, including the solvent energy and polarization effect, contributes 5.3 kcal mol^−1^ to free energy barrier height, and 27.1 kcal mol^−1^ to reaction energy.

To the best of our knowledge, so far there have been no experimental results of the reaction barrier heights available in aqueous solution to compare with our calculated one. Nonetheless, we can justify the accuracy of our calculated free energy barrier height by computing the free energy barrier height in aqueous solution based on gas-phase reaction profile and free energies of solvation.

**Figure 5:**
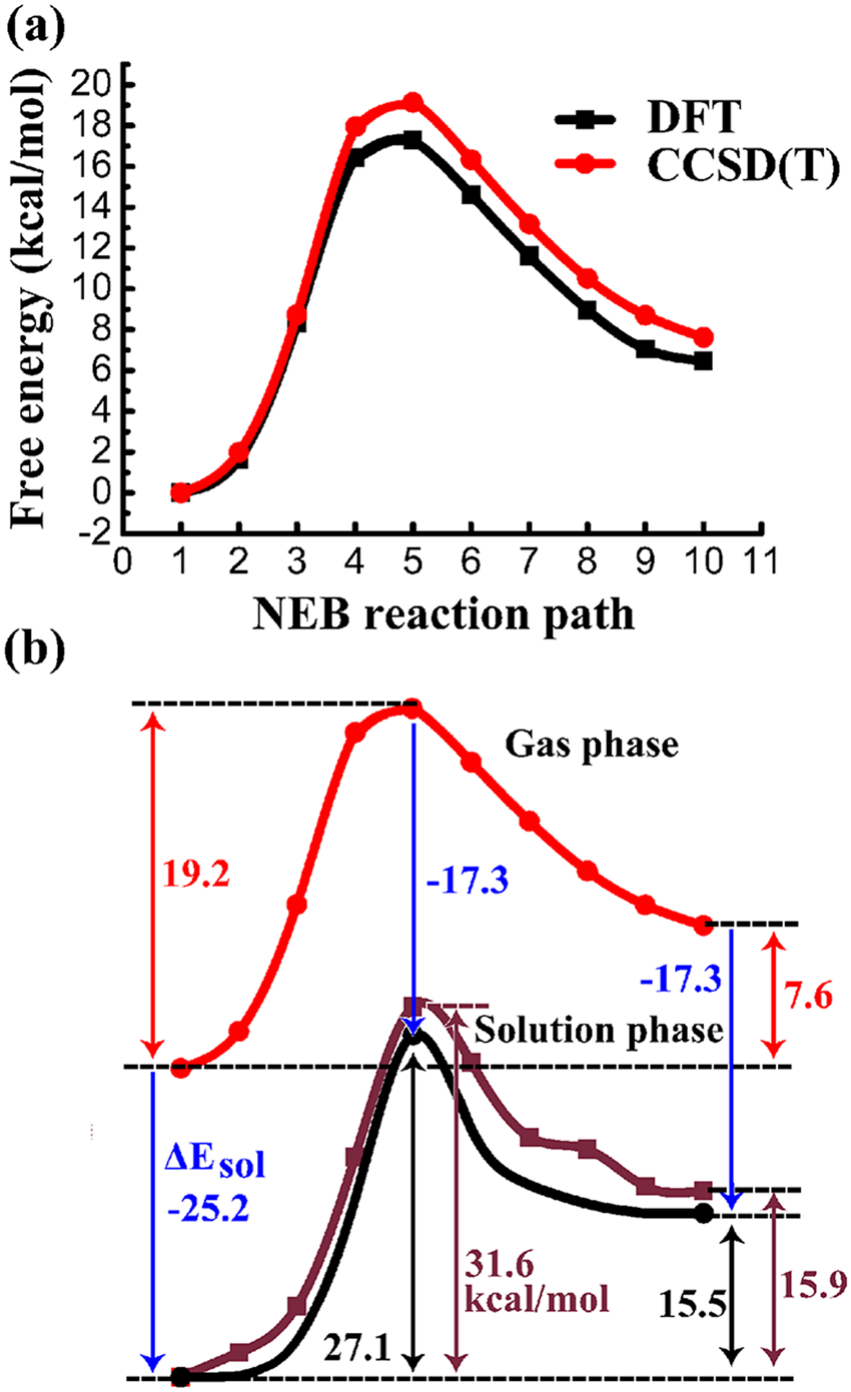
Reaction pathways along the NEB reaction path and comparison of reaction profiles. (**a**) Minimum energy path at DFT and CCSD(T) levels of theory with the reactant state as a reference point in gas phase, (**b**) Schematic plot of the comparison between the estimated reaction profile (the black curve obtained based on the gas-phase reaction path and free energies of solvation) and our calculated one at the CCSD(T)/MM level of theory (brown) in aqueous solution. The free energies of solvation from gas phase to solution phase are labeled in blue, and the estimated free energy of reaction and barrier height in solution, are labeled in black.

First, we calculated the NEB reaction pathway in gas phase using the multi-level QM method similar as we did in the solution. Figure 5a shows the gas-phase reaction pathway for the ring opening of 8-OHGrad at the DFT/B3LYP/6-311++G** and CCSD(T)/6-311++G** levels of theory with 10 beads. The structure of transition state in gas phase has been shown in Fig. 2b, which was confirmed by numerical frequency calculations with a single imaginary frequency of 236.2*i* cm^−1^. The energy barrier is 17.3 kcal mol^−1^ under DFT level of theory, while it is 19.2 kcal mol^−1^ under CCSD(T) level of theory. The CCSD(T) results, 19.2 kcal mol^−1^ for barrier height and 7.6 kcal mol^−1^ for reaction energy, show good agreement with the ones by Chaban *et al*.^41^, 20.8 kcal mol^−1^ for barrier height and 9.7 kcal mol^−1^ for reaction energy.

Second, we employed the conductor-like polarizable continuum model (CPCM)^50^ in GAUSSIAN09 software^51^ to calculate the free energies of solvation of the reactant complex, transition state and product complex. With the CPCM self-consistent reaction field (SCRF) calculations at B3LYP/aug-cc-pVDZ level of theory and a dielectric constant of 78.36 of aqueous solution, the free energies of solvation of reactant complex, transition state and product complex were obtained at −25.2 kcal mol^−1^, −17.3 kcal mol^−1^ and −17.3 kcal mol^−1^, respectively.

Third, based upon the reaction path obtained in the gas phase and the free energies of solvation obtained above, we can calculate the PMF in aqueous solution based on the gas-phase profile, then compare it with our calculated CCSD(T)/MM results with the explicit SPC/E water model. Figure 5b shows that the free energy barrier obtained using the gas-phase data is at 27.1 kcal mol^−1^, which agrees very well with our calculated value, 28.8 kcal mol^−1^ under the DFT/MM level of theory, also agrees with the 31.6 kcal mol^−1^ under the CCSD(T)/MM level of theory. The reaction energy obtained using the gas phase data is 15.5 kcal mol^−1^, showing a good agreement with our calculated values, 13.7 kcal mol^−1^ under the DFT/MM and 15.9 kcal mol^−1^ under CCSD(T)/MM level of theory.

We noticed there is a large gap of the free energy barrier height between our CCSD(T)/MM result (31.6 kcal mol^−1^) with an explicit SPC/E water model and the one by Munk *et al*. (19.5 kcal mol^−1^) with the IEF-PCM solvation model at DFT/B3LYP level of theory^33^. We think the free energy barrier obtained by Munk *et al*. might be too low for the ring opening process. Because the gas-phase reaction barrier we obtained here, 19.2 kcal mol^−1^, has a very good agreement with the one, 20.8 kcal mol^−1^, calculated by Chaban *et al*.^41^ from their *ab initio* multiconfigurational MCSCF/DZP/MRPT2 study; moreover, the free energy barrier height in aqueous solution obtained using the above gas-phase data is at 27.1 kcal mol^−1^, which is much larger than the one 19.2 kcal mol^−1^ by Munk *et al*., but agrees very well with our calculated value, 28.8 kcal mol^−1^ under the DFT/MM level of theory, also agrees with the one 31.6 kcal mol^−1^ under the CCSD(T)/MM level of theory. Therefore, we think the free energy barrier in aqueous solution obtained by Munk *et al*. might be too low for this reaction.

### Reaction rate constant

Considering that there is no experimental measurement of rate constants for the ring-opening reaction of 8-OHGrad to formimidic acid radical both in gas phase and in aqueous solution, we estimated the reaction rate constants using the thermodynamics equation^52^ of the transition state theory

1 $$k=Aexp(-\frac{{\rm{\Delta }}{W}_{a}^{\ddagger}}{RT})$$

where *A* is defined as (*k*_*B*_ *T*/*h*)·(*RT*/*P*), and *k*_*B*_, *h*, *R*, *P* and ${\rm{\Delta }}{W}_{a}^{\ddagger}$ represent the Boltzmann constant, Plank constant, gas phase constant, standard state pressure and the barrier heights in the gas phase and solution, respectively. Based on the barrier height of 19.2 kcal mol^−1^ under CCSD(T) level of theory in the gas phase and 31.6 kcal mol^−1^ under CCSD(T)/MM level of theory in aqueous solution, the rate constants in gas phase is about ~10^−21^ cm^3^/molecule/s and about ~10^−30^ cm^3^/molecule/s in the solution phase. This indicates the rate constant in solution is about 9 orders of magnitude smaller than in gas phase. In other words, the presence of water environment greatly reduced the reactivity.

## Summary and conclusions

Combining multi-level quantum mechanics theories and molecular mechanics (ML-QM/MM)^43–45^ with the explicit SPC/E^42^ solvent model, we studied the ring-opening mechanism of 8-OHGrad to formimidic acid radical both in gas phase and in aqueous solution. The presence of solvent greatly affects the structures of stationary points in aqueous solution, especially torsion angles. Detailed ring-opening reaction mechanism is obtained, showing a process of the gradually breaking of C8-N9 process. Under the CCSD(T) and CCSD(T)/MM levels of theory, the free energy barrier heights are 19.2 kcal mol^−1^ in gas phase and 31.6 kcal mol^−1^ in aqueous solution. Our calculated barrier height in the gas phase shows very good agreement with the earlier *ab initio* study by Chaban *et al*.^41^, and our calculated barrier height in aqueous solution is consistent with the one calculated based on the gas-phase profile and free energies of solvation, which indicates the one calculated by Munk *et al*. using IEF-PCM model might be too low. The solvent plays an important role in the PMF in aqueous solution by contributing 5.3 kcal mol^−1^ to free energy barrier height, and 27.1 kcal mol^−1^ to reaction energy.

Except for our investigations on the ring-opening mechanism of 8-OHGrad system, there are various paths for the guanine lesion of DNA. Their detailed reaction mechanism, accurate free-energy barrier heights and reaction energies both in the gas phase and in aqueous solution need to be further studied.

## Methods

In this paper, we combined multi-level quantum mechanics theories with molecular mechanics (ML-QM/MM)^43–45^ to study the direct ring opening process of 8-OHGrad system in water. We treated the solute 8-OHGrad as the QM region described using the multi CCSD(T), DFT and electrostatic potential (ESP)^53^ levels of theory during different stages of calculation, and treated water solution as the classical MM region. The potential energy of the whole reaction system can be expressed as,

2 $${V}_{potential}={V}_{qm}+{V}_{qm/mm}+{V}_{mm}$$

*V*_*qm*_ represents the potential of QM region with the same expression as in the gas phase and *V*_*mm*_ is the molecular mechanical energy of the MM region. The middle term, *V*_*qm*/*mm*_, is the coupling potential term representing van der Waals interactions, nuclear solute-solvent bond interactions, and the electrostatic interactions between the QM and MM regions.

A direct CCSD(T)/MM computation on the PMF is still too expensive to be carried out. By employing multi-level quantum theories, including the ESP, DFT and CCSD(T), for the QM region, we started from the low level, a relatively inexpensive DFT/MM level of theory, then shifted the calculation to the CCSD(T)/MM level of theory to achieve the more accurate PMF. Thus, the PMF under the CCSD(T)/MM level of theory was obtained^43^,

3 $${\rm{\Delta }}{W}_{AB}^{CC}=({\rm{\Delta }}{W}_{AA}^{CC\leftarrow DFT}+{\rm{\Delta }}{W}_{BB}^{CC\leftarrow DFT})+({\rm{\Delta }}{W}_{AA}^{DFT\leftarrow ESP}+{\rm{\Delta }}{W}_{BB}^{DFT\leftarrow ESP})+{\rm{\Delta }}{W}_{AB}^{ESP}$$

Here the last term, ${\rm{\Delta }}{W}_{A,B}^{ESP}$, is the solvent contribution to the PMF. The first two terms in parentheses stand for free energy difference of shifting the level from ESP to DFT and from DFT to CCSD(T) at the fixed solute configurations.

In this work, for the DFT level of theory calculation, we carried out two types of DFT levels of theory calculations: one used the aug-cc-pVDZ basis set, the other used 6–311++G** basis set, both with B3LYP functional. However, for the aug-cc-pVDZ basis, the calculation on the CCSD(T) level of theory requires huge CPU memories and our computation resources cannot fulfill this requirement, thus we have to settle on the 6-311++G** basis set in order to obtain the PMF on the CCSD(T) level of theory. Nevertheless, *ab initio* studies show that the DFT/B3LYP/6-311++G** combination gives consistently reliable geometries and conformationally dependent energies for carbohydrates^54–59^. Therefore, two kinds of DFT/B3LYP calculations with aug-cc-pVDZ basis and 6-311++G** basis were performed on the PMF calculations, and the results based on the two different DFT calculations are compared with each other; however, only the DFT/B3LYP/6-311++G** results were used to be shifted to obtain the PMF at the CCSD(T) level of theory.

The solute of the 8-OHGrad as the QM region was embedded in a 37.5 Å cube box consisting of 1752 water molecules described with an explicit SPC/E^42^ water model. The cutoff radius around the QM region is 15 Å, in which the 8-OHGrad interacts with water molecules *via* bonded interactions, electrostatic interactions, and van der Waals interactions. Outside the cutoff radius, there are only coulombic interactions between the MM charges and the QM ESP charges. The van der Waals parameters of the QM region were obtained from standard Amber force field^60^.
